# CI Therapy is Beneficial to Patients with Chronic Low-Functioning Hemiparesis after Stroke

**DOI:** 10.3389/fneur.2014.00204

**Published:** 2014-10-20

**Authors:** Annette Sterr, Darragh O’Neill, Philip J. A. Dean, Katherine A. Herron

**Affiliations:** ^1^School of Psychology, University of Surrey, Guildford, UK; ^2^School of Life and Medical Sciences, University College London, London, UK; ^3^Pain Management Centre, National Hospital for Neurology and Neurosurgery, University College London, London, UK

**Keywords:** arm, upper limb, shaping training, low-functioning, constraint, fatigue

## Abstract

CI therapy is effective in patients with relatively good levels of residual arm function but its applicability to patients with low-functioning hemiparesis is not entirely clear. In the present study, we examined the feasibility and efficacy of the CI therapy concept in patients with very limited upper arm function prior to treatment, and further tested how the length of daily shaping training and constraining the good arm affects treatment outcome. In a baseline-controlled design, 65 chronic patients were treated with 2 weeks of modified CI therapy. Patients were randomly allocated to four treatment groups receiving 90 or 180 min of daily shaping training applied with or without constraint, respectively. Outcome was measured through the Reliable Change Index, which was calculated for parameters of motor function, health, and psychological wellbeing. Follow-up data were collected at 6 and 12 months. Two analyses were conducted, a whole-group analysis across all 65 participants and a sub-group analysis contrasting the four treatment variants. The whole-group analysis showed a significant treatment effect, which was largely sustained after 1 year. The sub-group analysis revealed a mixed picture; while improvements against the baseline period were observed in all four subgroups, 180 min of daily shaping training coupled with the constraint yielded better outcome on the MAL but not the WMFT, while for 90 min of training the level of improvement was similar for those who wore the constraint and those who did not. Together these results suggest that, at least in those patients available for follow-up measures, modified CI therapy induces sustained improvements in motor function in patients with chronic low-functioning hemiparesis. The absence of clear differences between the four treatment variants points to a complex relationship between the length of daily shaping training and the constraint in this patient group, which is likely to be mediated by fatigue and/or compliance with the constraint.

## Introduction

Eighty-five percent of stroke survivors sustain upper limb hemiparesis (1). Several systematic reviews [e.g. Ref. (2, 3)] suggest that constraint-induced movement therapy (CI therapy), and modified versions thereof, achieve sustained improvements of upper limb function. While the evidence-base for CI therapy is strong, it is primarily relevant to patients with relatively good levels of residual recovery, with Taub himself estimating that about 20–25% of patients meet the minimum motor criteria for participation in the signature CI therapy intervention (4). This confinement to patients with relatively good residual recovery is one of the barriers to the implementation of CI therapy into clinical practice (5, 6). The focus of rehabilitation research on patients with better residual hand function is not unique to CI therapy, but reflects a trend in contemporary motor rehabilitation research (7, 8). Generally, treatment options for patients with poorer recovery are limited and the available evidence base is relatively weak (9, 10).

CI therapy is theoretically grounded and proposes tangible treatment mechanisms. It is designed to deliver improved motor function as well as changes in habitual motor behavior. The latter is achieved (i) through intensive paretic arm training, (ii) constraining the non-paretic arm, and (iii) by considering the psychological aspects of the treatment process (11, 12). Because of these credentials, the CI therapy concept is worthy of further exploration in more severely affected patients. However, its application in patients with limited residual ability poses challenges. For example, when residual ability is poor, everyday activities become very difficult, if not impossible, when the non-paretic arm is constrained. Patients might, therefore, struggle to maintain the level of independence that they are accustomed to while undergoing the CI therapy intervention. Moreover, by forcing the use of the paretic arm while restricting the non-paretic arm, the intervention is likely to expose the coping mechanisms and avoidance behaviors patients may have adopted in response to their compromised abilities. One might, therefore, argue that the constraint condition forces patients to confront their disability in a rather harsh way, and hence question the appropriateness of its use. On the other hand, evidence suggests that the constraint is an effective tool in fostering real-world treatment benefit (4), which may well outweigh its negative aspects and hence warrant, if not mandate its use. Investigating the feasibility of constraining the non-paretic hand, and the treatment benefit it promotes, is therefore required.

Another aspect of the CI therapy application in less well-recovered patients is that of treatment intensity. Previous studies have demonstrated that treatment regimes with longer training intervals induce greater benefit (13). However, it is also conceivable that longer training times might be less effective if patients become fatigued and tired. Protocols with shorter daily training sessions might, therefore, be equally or even more effective than longer training sessions in patients with greater motor limitations.

There is a wealth of literature on CI therapy and related protocols [e.g., Ref. (2, 3, 14, 15)]. However, to the best of our knowledge few published studies have attempted to apply CI therapy to patients not fulfilling the minimum motor criteria specified by the CI therapy signature intervention, and none have examined directly the benefit of constraining the non-paretic arm in relation to treatment intensity. Based on our previous work (16), the present study, therefore, examined the effects of non-paretic arm constraint (constraint vs. no constraint) and amount of daily training (180 vs. 90 min), as well as their interaction. Similar to the CI therapy signature intervention, a treatment contract was employed to ensure compliance and engagement. Treatment effects were determined through measures of motor function (primary outcomes), as well as healthy and psychological wellbeing (secondary outcomes).

## Methods

### Participants

**Figure 1:**
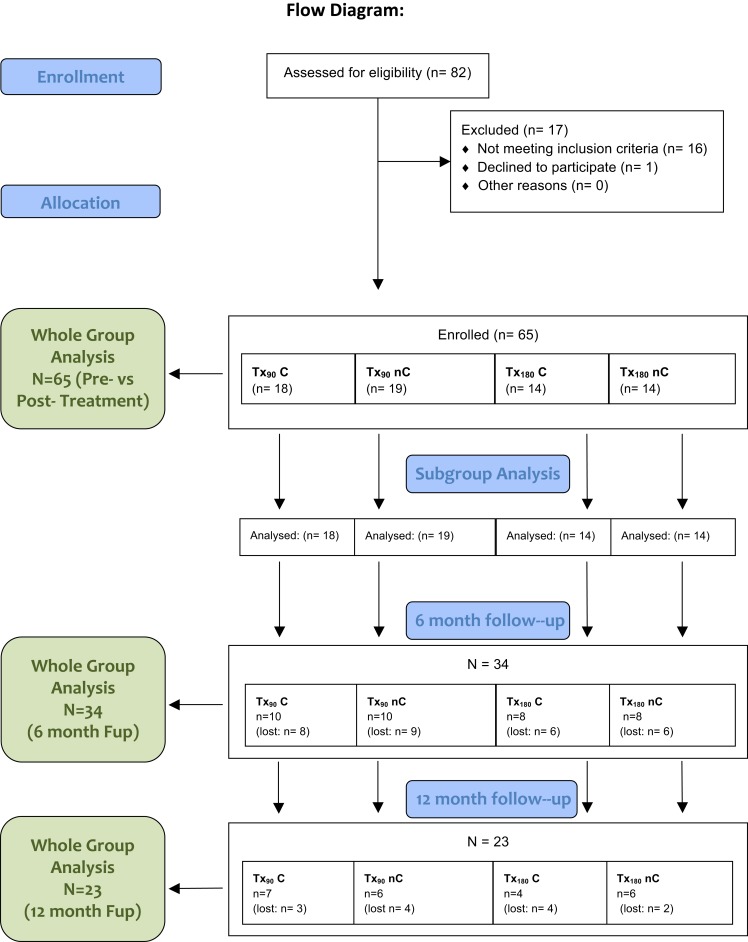
**Summary of the trial and group allocation**. Note that (1) all patients received the allocated treatment, (2) the *n* for “patients lost” refers to the number of participants having completed the MAL, (3) for attending the follow-up did not necessarily completed all tests; a full breakdown of *n* per test is given in the supplementary materials, and (4) the cause for drop out were difficulties/unwillingness to traveling to the University (many patients had come from further afield).

Patients were recruited via local general practitioners (GPs), self-help groups, and newspaper advertisements. Eighty-two patients with first-ever stroke and chronic hemiplegia for a minimum of 1 year were screened. Sixty-five of these participants (mean age was 54.4 ± 1.5 years; 38 male and 27 female; 36 left and 29 right hemiparesis; chronicity = 4.3 ± 0.4 years with range = 1–14.9 years) participated in the study and complete the actual treatment phase. Due to drop outs, the N reduced to 34 at the 6-months follow-up and 23 at the 12-months follow-up (see Figure 1 for summary). Patients were recruited on the basis of the presentation of their motor deficits rather than lesion location. Confirmation of a unilateral thalamic or cortical stroke was obtained from the GP, but no specific lesion information was available to us.

Low-functioning hemiparesis was defined as a minimum motor criterion comprising the ability to produce a voluntary movement with any part of the hand, a finger, or the wrist no matter how small. Patients who met or exceeded Taub’s criterion of 20° wrist and 10° finger extension were excluded. Further exclusion criteria comprised seizures 6 months prior to participation, frequent falls, severe aphasia, a history of major secondary medical or mental health conditions, and a Mini-Mental State Score <24. All patients were community dwelling and lived either with family or a carer.

The study took place in a laboratory housed at the School of Psychology within the University of Surrey. The study protocol was approved by the NHS Surrey Research Ethics Committee and the Ethics Committee of the University of Surrey. Written informed consent and GP assent were obtained prior to participation.

### Intervention

Patients were randomly allocated to four versions of modified CI therapy (13, 16, 17), comprising daily training of either 180 or 90 min per day (Tx_180_ or Tx_90_) and the presence or absence of the constraint (C or nC, respectively), resulting in the groups Tx_90_ C (*N* = 18), Tx_90_ nC (*N* = 19), Tx_180_ C (*N* = 14), and Tx_180_ nC (*N* = 14; see Table 1 for demographic details). Patients were consecutively allocated to the groups in the order of intake. Randomization was not blinded. Treatment was provided daily for 10 consecutive days spread over a fortnight.

**Table 1:** **Demographic characteristics or the treatment groups**.

Therapy	Constraint	N	Age	Gender (M/F)	Hemiparesis (L/R)	Chronicity (years)
Tx_90_	N	19	55.8 ± 2.6	7/12	12/7	4.5 ± 0.8
	Y	18	57.3 ± 2.6	12/6	10/8	4.4 ± 0.6
Tx_180_	N	14	56.4 ± 3.1	10/4	9/5	3.4 ± 0.9
	Y	14	46.9 ± 3.5	9/5	5/9	4.6 ± 1.2
		65	54.4 ± 1.5	38/27	36/29	4.3 ± 0.4

*Gender (M/F), male/female; hemiparesis (L/R), side (Left/Right) of hemiparetic limb; chronicity, time since stroke*.

The paretic arm training used the principles of shaping training (12), with tasks individually adapted to accommodate the patients’ level of ability. Training was complemented by a treatment contract, brief daily problem solving sessions, and a diary in which patients noted the use of the constraint (if applicable) and their activities outside the treatment setting.

Patients in the two constraint groups (Tx90C, Tx180C) were asked to wear the constraint at home as specified in the treatment contract. The latter was tailored to participants’ ability, and defined situations in which the constraint could be worn safely. This was reviewed at the beginning of every session as motor ability improved, or as difficulties arose.

### Testing procedure

A baseline-controlled cross-over design with five time points was used: baseline (two weeks prior to treatment), pre (start of treatment), post (end of treatment), and follow-up measures at 6 and 12 months (Fup6, Fup12). In these testing sessions, data on motor function and psychological wellbeing were acquired through a range of tests comprising the Frenchay Arm Test [FAT (18)], an adapted version of the graded version of the Wolf Motor Function Test [WMFT (19); http://www.uab.edu/citherapy/images/pdf_files/CIT_Training_WMFT_Manual.pdf], the Nine-Hole Peg Test [NHPT (20)], and the Motor Activity Log [MAL (21)]. In the same sessions, data on general health and psychological wellbeing were acquired using the Short Form 36 [SF36 (22)], Stroke Impact Scale [SIS (23)], Hospital Anxiety and Depression Scale [HADS (24)], and Visual Analog Mood Scale [VAMS (25)]. Further details of the tests are provided in the legend of Table 2 as well as in the Supplementary Materials.

**Table 2:** **Summary table for whole-group analysis statistics**.

Test	Pre-post	Post-Fup6	Fup6-Fup12
FAT	*t* = 3.7_(64)_, *p* < 0.001, *d* = 0.61	*t* = −1.7_(30)_, *p* < 0.095, *d* = 0.31	*t* = 1.2_(18)_, *p* < 0.2, *d* = 0.28
WMFT TT	*t* = −2.9_(64)_, *p* < 0.01, *d* = 0.35	*t* = 0.7_(30)_, *p* < 0.5, *d* = 0.12	*t* = −1.5_(19)_, *p* < 0.1, *d* = 0.34
WMFT FA	*t* = 8.0_(64)_, *p* < 0.001, *d* = 0.99	*t* = 0.5_(30)_, *p* < 0.9, *d* = 0.03	*t* = 3.1_(19)_, *p* < 0.007, *d* = 0.68
MAL AoU	*t* = 9.6_(64)_, *p* < 0.001, *d* = 1.19	*t* = −2.4_(33)_, *p* < 0.025, *d* = 0.4	*t* = −0.3_(22)_, *p* < 0.8, *d* = 0.06
MAL QoM	*t* = 11.1_(64)_,*p* < 0.001, *d* = 1.38	*t* = −1.8_(33)_, *p* < 0.07, *d* = 0.32	*t* = −0.5_(22)_, *p* < 0.6, *d* = 0.11
NHPT S	*t* = 4.4_(62)_, *p* < 0.001, *d* = 0.46	*t* = −0.2_(29),_ *p* < 0.8, *d* = 0.04	*t* = 0.8_(18)_, *p* < 0.4, *d* = 0.18
NHPT L	*t* = 1.6_(63),_ *p* < 0.1, *d* = 0.2	*t* = 0.9_(29)_, *p* < 0.4, *d* = 0.17	*t* = 0.4_(18)_, *p* < 0.7, *d* = 0.1
SIS Tot	*t* = 6.4_(63)_, *p* < 0.001, *d* = 0.8	*t* = 1.4_(32)_, *p* < 0.2, *d* = 0.25	*t* = −1.1_(22)_, *p* < 0.3, *d* = 0.23
SIS Phys	*t* = 7.0_(63)_, *p* < 0.001, *d* = 0.87	*t* = 0.8_(32)_, *p* < 0.5, *d* = 0.13	*t* = −1.6_(22)_, *p* < 0.1, *d* = 0.32
SF36 P	*t* = 0.8_(60)_, *p* < 0.4, *d* = 0.11	*t* = 1.9_(31)_, *p* < 0.074, *d* = 0.33	*t* = −0.9_(21)_, *p* < 0.4, *d* = 0.2
SF36 M	*t* = 0.7_(60)_, *p* < 0.5, *d* = 0.09	*t* = 1.9_(31)_, *p* < 0.071, *d* = 0.33	*t* = 0.02_(21)_, *p* < 0.99, *d* < 0.01
HADS A	*t* = −2.4_(60)_, *p* < 0.05, *d* = 0.30	*t* = −2.3_(30)_, *p* < 0.032, *d* = 0.4	*t* = 1.8_(20)_, *p* < 0.084, *d* = 0.4
HADS D	*t* = −0.6_(60)_, *p* < 0.6, *d* = 0.08	*t* = −2.9_(30)_, P < 0.007, *d* = 0.52	*t* = 0.6_(20)_, *p* < 0.6, *d* = 0.13
VAMS P	*t* = 0.9_(59)_, *p* < 0.4, *d* = 0.12	*t* = 1.4_(31)_, *p* < 0.2, *d* = 0.26	*t* = −0.4_(21)_, *p* < 0.7, *d* = 0.09
VAMS N	*t* = 0.5_(59)_, *p* < 0.6, *d* = 0.06	*t* = −0.1_(31)_, *p* < 0.9, *d* = 0.02	*t* = 0.7_(21)_, *p* < 0.5, *d* = 0.15

*Gray boxes highlight significant changes*.

*The acronyms are as follows: FAT, Frenchay Arm Test; WMFT FA, Wolf Motor Function Test Functional Ability; WMFT TT, Wolf Motor Function Test Time Taken; MAL AoU, Motor Activity Log Amount of Use; MAL QoM, Motor Activity Log Quality of Movement; NHPT S, Nine-Hole Peg Test Small; NHPT L, Nine-Hole Peg Test Large; SIS Tot, Stroke Impact Score Total; SIS Phys, Stroke Impact Score Physical subscale; SF36, Short Form 36; HADS A, Hospital Anxiety and Depression Scale Anxiety subscore; HADS D, Hospital Anxiety and Depression Scale Depression subscore; VAMS, Visual Analog Mood Score with VAMS, P, positive; and VAMS N, negative*.

### Analysis and statistics

Demographic data were analyzed for the factor TREATMENT GROUP using one-way ANOVAs or chi-square tests as appropriate.

For the main analysis of primary and secondary outcome, longitudinal treatment benefits were evaluated through the reliable change index approach [RCI (26, 27)]. This measure determined the standard error of difference from the variation in outcome indices from baseline to pre-therapy interval. The standard error of difference is used in RCI analysis as a measure of baseline fluctuation from which reliable, significant change during and after the treatment phase can be calculated. This method is described in detail in the Supplementary Materials; the raw scores are listed in Table S2 in Supplementary Material.

For each outcome measure, RCI was calculated for the therapy interval (Pre-post; termed *treatment effect* in the manuscript), and for changes over the follow-up period between post therapy and 6 months after therapy (Post-Fup6), and between 6 and 12 months post therapy (Fup6-Fup12). In this way, *treatment effect* expressed as RCI represents a measure of significant change with treatment corrected for baseline fluctuations; while *follow-up* measures the longevity of these treatment effects in relation to baseline fluctuations.

The RCI scores were subsequently analyzed in two ways. Firstly, to test whether the CI therapy concept improved motor function in the chronic state, a whole-group analysis across all 65 participants was conducted using one-sample *t*-tests (versus zero, representing no reliable change). To examine, the relative difference between the four treatment variants factorial 2 × 2 ANOVAs with CONSTRAINT condition (C/nC) and TRAINING INTENSITY (T_180_/T_90_) were calculated for each outcome parameter, respectively. Effect sizes were calculated using Cohen’s *d* for the whole-group analysis and eta squared $\left(\eta_{p}^{2}\right)$ for sub-group analysis. Eta squared was used in the ANOVA analysis to determine the proportion of variance in outcome measure attributed to the each therapy modification. Effect sizes were categorized using the following criteria of small $\left(d=0.2;\;\eta_{p}^{2}=0.01\right)$, medium $\left(d=0.5;\;\eta_{p}^{2}=0.06\right)$, and large $\left(d=0.8;\;\eta_{p}^{2}=0.14\right)$. Because of large drop out in the follow-up period, the follow-up analysis was only sensibly for the whole-group since as the cell size became too small for the sub-group analysis. However, for completion, the sub-group analysis is made available in Table S1 in Supplementary Materials).

Finally, the association between primary (motoric) and secondary (health and psychological wellbeing) outcomes after CI therapy was explored through regressions between the treatment effect RCIs for the primary outcome measures (FAT, WMFT, NHPT, MAL; primary outcome measures) and treatment effect RCIs of the secondary outcome measures (SIS, SF36, HADS, VAMS).

The results below will first summarize the findings for the group level including treatment effect and follow-up, followed by the sub-group analysis (treatment effect only) and correlations.

## Results

### Demographic data

There were no significant differences between the four treatment groups in terms of baseline WMFT-FA [*F*(3,56) = 1.2, *p* = 0.3], FAT [*F*(3,56) = 0.2, *p* = 0.9], side of hemiparesis [χ^2^ (3, *N* = 65) = 3.1; *p* = 0.4], chronicity [*F*(3,64) = 0.3, *p* = 0.8], gender [χ^2^ (3, *N* = 65) = 5.3; *p* = 0.2], and age [*F*(3,64) = 2.5, *p* = 0.07]. The patients were also evenly distributed between groups such that there were equal proportions wearing a constraint in the 90 and 180 min therapy groups, and equal numbers with the greatest training intensity in the constraint and no constraint groups [χ^2^ (1, *N* = 65) = 0.1; *p* = 0.9].

### Whole-group analysis

#### Treatment effect

**Figure 2:**
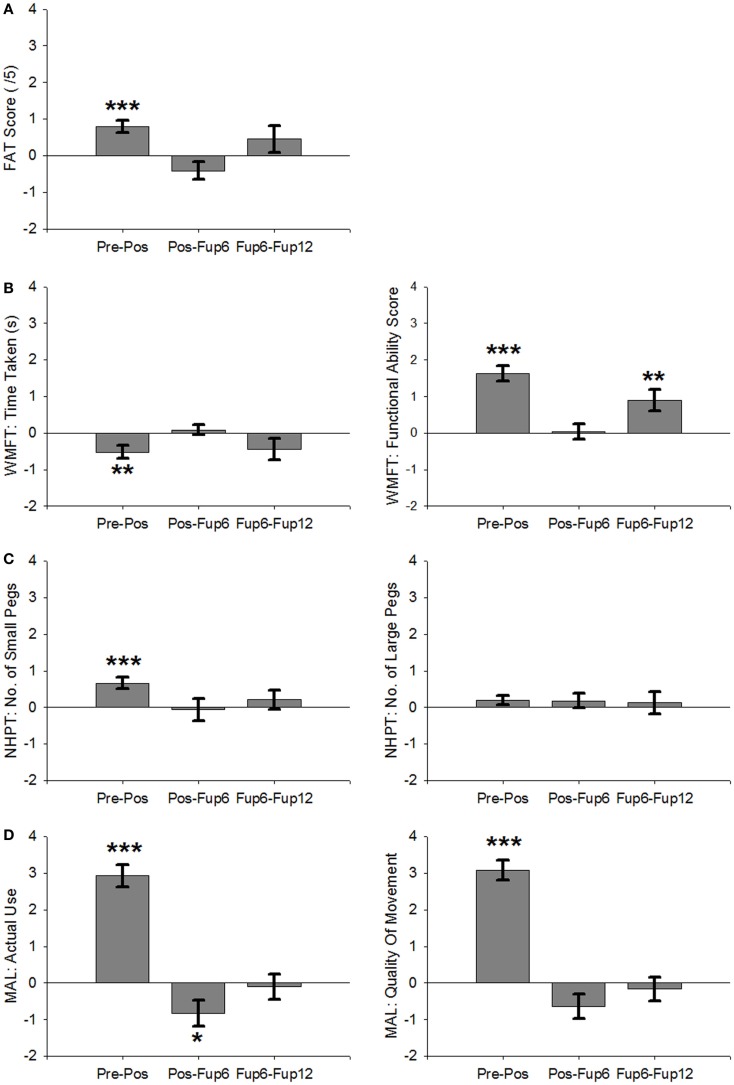
**Average RCI across all participants over the course of therapy and follow-up**. **(A)** Frenchay Arm Test. **(B)** Wolf Motor Function Test: Time Taken (left) and Functional Ability Scale (right). **(C)** Nine-Hole Peg Test: number of small pegs (left) and large pegs (right). **(D)** Motor Activity Log: Amount of Use (left) and Quality of Movement (right). Error Bars are SEM. ***p < 0.001, **p < 0.01, *p < 0.05.

The primary outcome measures indicated significant improvements in motor function (Figure 2) with large effects on both MAL scales [AoU: *t*(64) = 9.6, *p* < 0.001, *d* = 1.19; QoM: *t*(64) = 11.1, *p* < 0.001, *d* = 1.38], and WMFT-FA [*t*(64) = 8.0, *p* < 0.001, *d* = 0.99], as well as a medium sized effect on the FAT [*t*(64) = 3.7, *p* < 0.001, *d* = 0.61], and small-to-medium effects on the NHPT_S [*t*(62) = 4.4; *p* < 0.001, *d* = 0.46], and the WMFT-TT [*t*(64) = −2.9, *p* = 0.01, *d* = 0.35] An improvement of small effect size was found for the NHPT_L, but this effect did not reach significance [*t*(63) = 1.6, *p* = 0.1, *d* = 0.2].

**Figure 3:**
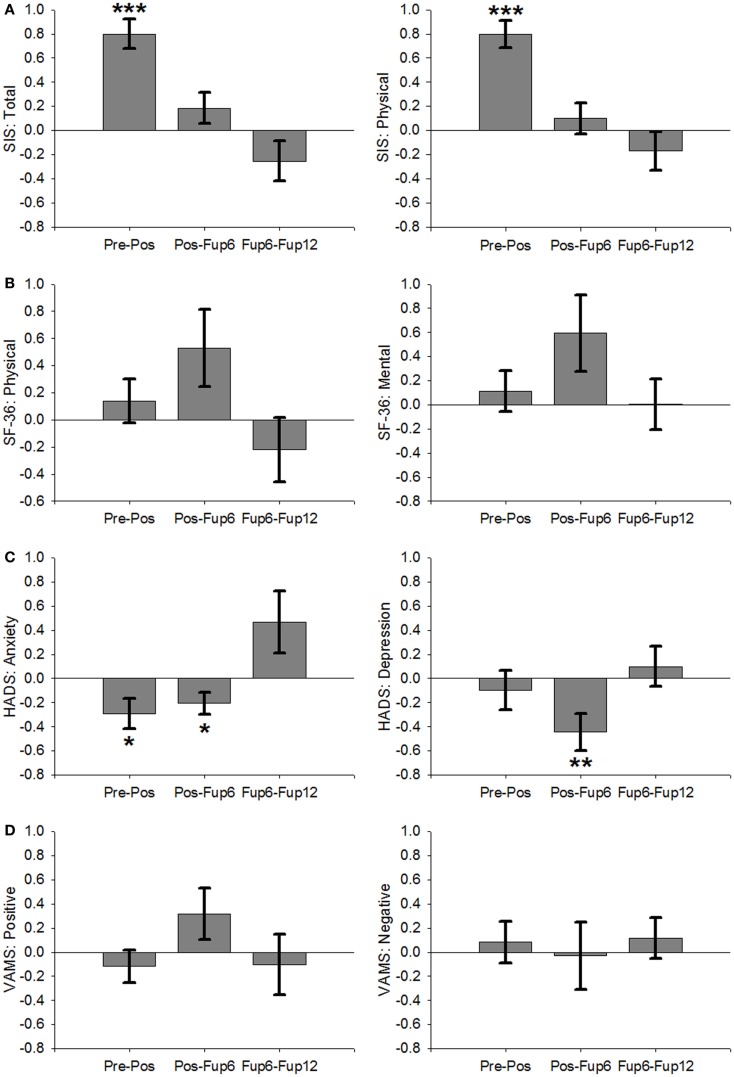
**Non-motoric measures across all participants over the course of therapy and follow-up period**. **(A)** Stroke Impact Scale: total (left) and physical subscale (right). **(B)** Short Form 36: physical total (left) and mental total (right). **(C)** Hospital Anxiety (left) and Depression (right) Scale. **(D)** Visual Analog Mood Score: positive mood (left) and negative mood (right). Error Bars are SEM. ***p < 0.001, **p < 0.01, *p < 0.05.

For the secondary outcomes improvements (Figure 3) were demonstrated by large effects on the physical subscale of the SIS [*t*(63) = 7.0, *p* < 0.001, *d* = 0.87] and the overall SIS score [*t*(63) = 6.4, *p* < 0.001, *d* = 0.80], as well as the anxiety subscale of the HADS [*t*(60) = −2.4, *p* = 0.05, *d* = 0.30]. No significant changes were found for the depression subscale of the HADS [*t*(60) = −0.6; *p* = 0.6, *d* = 0.08], the physical and mental subscales of the SF36 [*P*: *t*(60) = 0.8, *p* = 0.4, *d* = 0.11; *M*: *t*(60) = 0.7, *p* = 0.5, *d* = 0.09], or the VAMS [positive: *t*(59) = −0.9, *p* = 0.4, *d* = 0.12; negative: *t*(59) = 0.5, *p* = 0.6, *d* = 0.06].

#### Follow-up

The majority of tests for which a significant treatment effect was found did not change significantly over the 12-month follow-up period, indicating that the therapy-induced benefits were sustained over this period (see Table 2 as well as Figures 1 and 2).

However, a significant decline occurred in MAL-AoU scores from post to Fup6 [*t*(33) = −2.4, *p* = 0.025, *d* = 0.4], although the effect size of this decline was not as great as the effect size of the improvement during therapy (*d* = 1.19) suggesting that the amount of affected arm use at 6-month post treatment was still greater than during the 2-week baseline interval immediately preceding the intervention. Moreover, the decline did not continue between Fup6 and Fup12 [*t*(22) = −0.3, *p* = 0.8, *d* = 0.06]. For WMFT-FA scores improved from Fup6 to Fup12 [*t*(19) = 3.1, *p* = 0.007, *d* = 0.68], but showed no notable change from post to Fup6 [*t*(33) = 0.2, *p* = 0.9, *d* = 0.03]. Anxiety scores reduced further from post to Fup6 [*t*(30) = −2.3, *p* = 0.032, *d* = 0.4], but showed no significant change between Fup6 and Fup12 [*t*(20) = 1.8, *p* = 0.084, *d* = 0.4].

Outcome measures with no significant therapy effects remained unchanged over the follow-up period (see Table 2), except for depression scores, which reduced from post to Fup6 [*t*(19) = −2.9, *p* = 0.007, *d* = 0.52] and stayed unchanged Fup6–Fup12 [*t*(20) = 0.6, *p* = 0.6, *d* = 0.13].

### Sub-group analysis: Effects of training intensity and constraint on outcome

#### Treatment effect

**Figure 4:**
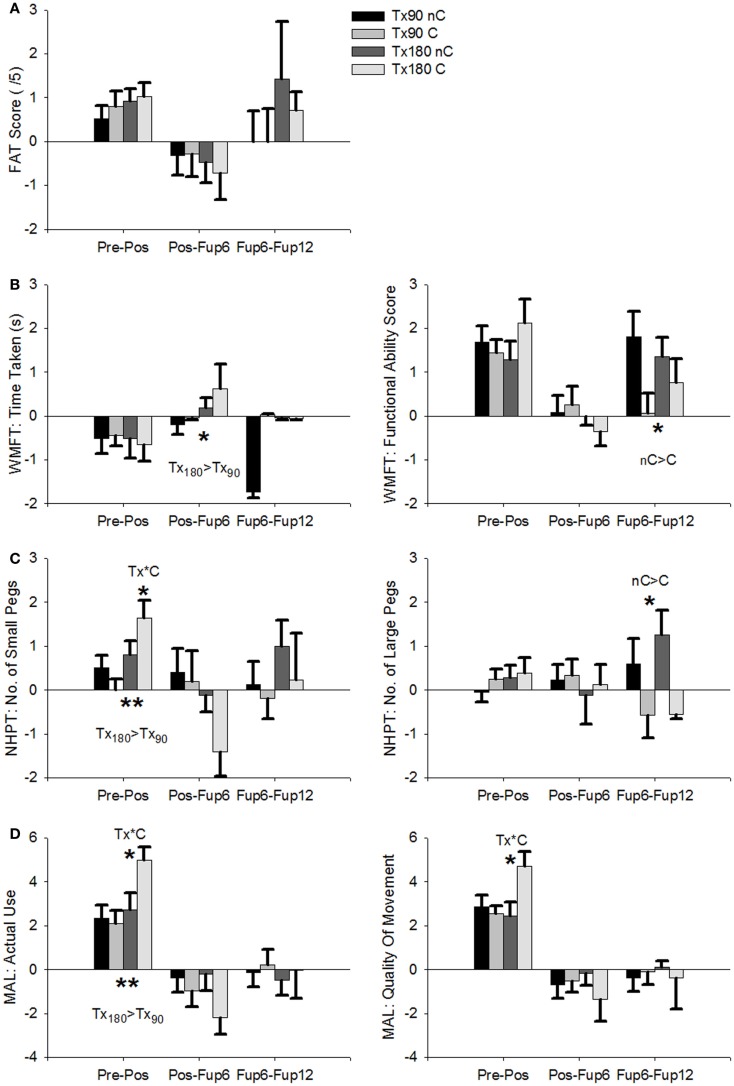
**Average RCI for each therapy group over the course of therapy and follow-up**. Tx_90_/Tx_180_: 90/180 min’s therapy; nC/C: No Constraint/With Constraint. **(A)** Frenchay Arm Test. **(B)** Wolf Motor Function Test: Time Taken (left) and Functional Ability Scale (right). **(C)** Nine-Hole Peg Test: number of small pegs (left) and large pegs (right). **(D)** Motor Activity Log: Amount of Use (left) and Quality of Movement (right). Error Bars are SEM. ***p < 0.001, **p < 0.01, *p < 0.05. Probability values indicate an interaction of treatment or follow-up and either constraint (C) or training intensity (Tx).

For the primary outcome parameters, the ANOVAs revealed a large effect of TRAINING INTENSITY on NHPT_S $\left[F\left(1,62\right)=12.7,\;p=0.001,\;\eta_{p}^{2}=0.18\right]$ and a medium-to-large effect on MAL AoU $\left[F\left(1,64\right)=8.3,\;p=0.005,\;\eta_{p}^{2}=0.12\right]$, with greater hours of training resulting in greater improvement (Figure 4). Interactions between TRAINING INTENSITY and CONSTRAINT were found for NHPT_S $\left[F\left(1,62\right)=5.9,\;p=0.018,\;\eta_{p}^{2}=0.09\right]$, MAL AoU $\left[F\left(1,64\right)=4.9,\;p=0.03,\;\eta_{p}^{2}=0.08\right]$, and MAL QoM $\left[F\left(1,64\right)=6.0,\;p=0.017,\;\eta_{p}^{2}=0.09\right]$, all with medium effect size. These interactions were due to differences in TRAINING INTENSITY between the two CONSTRAINT groups, with greater change observed in patients who wore the constraint and were in the Tx180C group compared to other treatment groups. None of the other motoric scores showed significant differences between the treatment groups.

For the secondary outcome parameters, no significant main effects or interactions were found except for SF36 physical, which showed greater improvement in patients using a CONSTRAINT $\left[F\left(1,60\right)=4.35,\;p=0.041,\;\eta_{p}^{2}=0.07\right]$, a medium-sized effect, despite the absence of significant improvement in this scale across all patients.

#### Follow-up

Because of substantive drop out, the group size is very small. The follow-up data for the sub-group comparison are, therefore, too weak to be fully reported. They are, however, summarized in the Supplementary Materials. Of note, the treatment effect of those patients available for follow-up testing and those dropping out after the post session was not significant for any of the motor variables or psychological measures.

### Relationship between primary (motor) and secondary (health/psychology) parameters

Regression analysis of treatment effect RCIs revealed that better MAL-AoU outcome was significantly associated with better outcome on general health and psychological wellbeing indices [*F*(8,58) = 3.4, *p* = 0.004]. More specifically, better outcome measured by MAL AoU was associated with better score on the SF36 Physical [β = 0.38, *t*(50) = 2.31, *p* = 0.025], as well as trend for SIS Total [β = 0.43, *t*(50) = 1.92, *p* = 0.061]. There were no other significant associations between motoric and non-motoric outcome.

## Discussion

The present study investigated the feasibility and efficacy of the CI therapy concept in patients with poorer residual recovery, and further explored how the two main treatment elements, constraint, and daily shaping training, affect treatment outcome. In alignment with CI therapy studies in higher functioning patients, a baseline-controlled paradigm with 2 weeks of baseline followed by 2 weeks of intervention was employed. The study was motivated by the assumption that these treatment elements not only interact with each other, but that this interaction may also be modulated by the severity of the motor deficit.

Confirming initial reports (28, 29) that positive outcomes can be achieved through CI therapy in patients with more severe hemiparesis, the data obtained through the whole-group analysis provides further and stronger evidence that the CI therapy approach is feasible and successful in patients with very poorer residual recovery. Significant benefits were observed across all patients on literally all motor outcomes, including those subjectively assessing real-world behavior and those assessing motor function through formalized tests. Moreover, these benefits were largely sustained over the 12-month follow-up period. This suggests that the CI therapy variants tested in this study promote sustained improvements of functional motor skills, and the use of these skills in everyday life, in patients with minimal ability. In addition, CI therapy had either positive or neutral effects on the patients’ wellbeing and mental health. This is an important finding, as it highlights the wider benefits on CI therapy, and further demonstrates that CI therapy has no adverse effects in patients with poor recovery.

With regards to the sub-group analysis of the four CI therapy variants tested in this study, the differences in outcome were relatively small and primarily affected the MAL. More specifically, using the constraint or having more/less shaping training did not significantly change the treatment outcome on either scale of the WMFT, or the FAT. In other words, the treatment benefit indexed by the WMFT subscales, MAL QoM, and FAT was equally strong in patients who wore the constraint and in those who did not, and in those who received 180 min of daily training and 90 min of daily training, respectively. This is an unexpected finding, not in the least since CI therapy studies on the relationship between treatment intensity and outcome suggest better outcomes for longer entities of daily training (13, 30). Furthermore, theoretical and empirical evidence on constraining the intact limb in animals implies that constraining this extremity is necessary to overcome the learned non-use phenomenon (22, 31), and promotes the implementation of newly learned skills from the therapy setting into everyday situations (17). This should translate into better treatment outcome. However, the findings of the present study do not conform to these assumptions. Rather, they suggest that only the combination of 180 min of training with the constraint produces improved outcome with regard to the subjective perception of everyday arm use and objective measures of fine finger movement. For 90 min of training on the other hand, wearing the constraint did not enhance the treatment benefit. Moreover, if patients received 180 min of training but did not wear the constraint, the treatment benefit was similar to those receiving only 90 min of daily training.

Of course, the findings reported here are based on relatively small sample sizes (*N* between 14 an 19 per group), and the result pattern observed here might be explained by poor test power. However, if the relative contribution of the constraint and amount of training were substantive, one would expect to see significant differences in smaller groups, in particular in groups with relatively homogenous demographics. The absence of strong and clear group differences between the CI therapy variants tested here, therefore, warrants further consideration. Moreover, the study suffered from substantive drop out and the notion that CI therapy achieves long-term improvements only holds for those patients who remained in the sample for the follow-up period. However, none of the motor or mood variable, or indeed age of chronicity, showed significant differences between the group of patients completing follow-up sessions and those dropping out after the post-training assessment. In addition, this study was conducted in the University and a number of patients came from far away, and had stayed in rented accommodation for the therapy session. Returning for a 1/2-day follow-up session was, therefore, not feasible for many of them. These logistical into considerations combined with the fact that no statistical difference existed between those who dropped out and those who did not indicates that the high drop out rate was caused by the study characteristics rather than the intervention *per se*.

We propose that the lack of substantive differences between the CI therapy variants might be explained by an interaction between the treatment mechanisms (constraint/training intensity) and the severity of the motor deficit. Thus, we suggest that the CI therapy intervention is physically and mentally tiring, and much more so for patients with minimal recovery. Their limited physical and cognitive capacity is likely to impact the way in which they are able to engage with the treatment, and possibly the efficacy of the treatment mechanisms as well. We assume that if the length of the training session exceeds this capacity, patients may become too fatigued to fully engage with the intervention. In contrast to high functioning patients, longer training sessions might therefore not necessarily be more effective than shorter training sessions in this patient group. On other words, it might be the case that at least for some patients, the additional treatment time provided for the 180 min group was rendered ineffective because patients had become too fatigued. Moreover, we speculate that adherence to the constraint condition is particularly challenging in patients with minimal recovery. Patients may find it difficult to pursue everyday activities when wearing the constraint and adherence might, therefore, have been quite varied across participants. However, because longer training sessions provide twice the contact time with the therapist, it might be that patients in this group could be motivated better to adhere to the constraint regime than patients with 90 min contact time. In addition, similar to the vast majority of published CI therapy studies, the present study captured the amount of time that the constraint was worn outside the therapy setting through diary entries. Not all participants completed the diary, and the entries varied substantively between 1 and 14 h supporting the idea that constraint adherence is a major factor to be considered in future studies.

Taken together our data suggest that the CI therapy concept is feasible and can effectively improve motor function in patients with low-functioning chronic hemiparesis. As such, the data expand the evidence-base for CI therapy to a wider group of patients and hence addresses one of the barriers to the clinical implementation of this intervention (5). However, our data also reveal that the components underpinning CI therapy (constraint, training intensity) may have different effects in patients with more severe hemiparesis. The findings highlight the need to further characterize the interaction between residual motor abilities and the treatment mechanisms with regards to adherence, motivation, and, most critically, fatigue. Larger trials that directly compare shorter and longer training times in conjunction with and without the constraint are necessary to create the evidence-base needed to optimize CI therapy for patients with varying degrees of residual function.

## Conflict of Interest Statement

The authors declare that the research was conducted in the absence of any commercial or financial relationships that could be construed as a potential conflict of interest.

## Supplementary Material

The Supplementary Material for this article can be found online at http://www.frontiersin.org/Journal/10.3389/fneur.2014.00204/abstract
