# Supplementary material for: CI Therapy is Beneficial to Patients with Chronic Low-Functioning Hemiparesis after Stroke
Source: Front Neurol. 2014 Oct 20;5:204. doi: 10.3389/fneur.2014.00204 (PMC4202624; doi:10.3389/fneur.2014.00204)
Supplement: Supplementary file 1 [file Data_Sheet1.PDF]

Supplementary Materials:

1. **Methods:**

Details of test battery:

Motor ability was recorded using the Frenchay Arm Test (FAT[19]; range: 0 to 5), the graded Wolf Motor Function Test (WMFT; comprising the Functional Ability Score [FAS[20](http://www.uab.edu/citherapy/images/pdf_files/CIT_Training_WMFT_Manual.pdf); modified range: 0 to 7 as listed below) and Time Taken [TT; median time in seconds]) and the Nine Hole Peg Test (NHPT[21]; using Large Pegs [NHPT L] or Small Pegs [NHPT S]. Subjective motor ability was measured with the Motor Activity Log (MAL; comprising Quality of Movement [QoM; range: 0 to 5] and amount of use [AoU[22]; range: 0 to 5]). The mean score per item/task was used in the analysis for WMFT FAS, MAL QoM and MAL AoU scores, whereas the median WMFT TT was used to reduce the effect of outliers. NHPT scores were the straight sum of the number of pegs moved.

Modified FA scale:

Level B:

0 = Does not attempt with involved arm
1 = Involved arm does not participate functionally; however, attempt is made to use the arm. In unilateral tasks the uninvolved extremity may be used to move the involved extremity
2 = Does, but requires assistance of uninvolved extremity for minor adjustments or change of position, or requires more than two attempts to complete, or accomplishes very slow
3 Does, but movement is performed slowly, and/or wit effect, and/or with excessive compensatory movements
Level A:

4* = Does, but requires assistance of uninvolved extremity for minor adjustments or change of position, or requires more than two attempts to complete, or accomplishes very slow

5* Does, but movement is performed slowly, and/or wit effect, and/or with excessive compensatory movements

6 = Does; movement is close to normal**, but slightly slower, may lack precision, fine coordination or fluidity

7 = Does; movement appears to be normal**

(*) The language of ratings 4 and 5 on LevelA is the same as for ratings 2 and 3 on level B and are applied to rating arm movement in the same manner. The difference is that the task is more difficult at Level A; subjects in effect, receive extra points for performing the Level A.

(**) For determination of normal, the uninvolved limb can be used for comparison, with premorbid limb dominance taken into consideration.

General aspects of health and the holistic impact of the stroke were assessed through the Short Form 36 (SF-36[30] Physical and SF-36 Mental subcales; range: 0 to 100) and the Stroke Impact Scale Version 2.0 (SIS[31] Total and SIS Physical; range 0 to 100). Mental health and mood were captured with the Hospital Anxiety and Depression Scale (HADS[32]; range: 0 to 21) and the Visual Analogue Mood Scale (VAMS[33] Positive [Energy, Tired] and VAMS Negative [Afraid, Confused, Sad, Angry, Tired, Tense]; range: 0 to 100). SF-36, SIS and VAMS scores were normalised across items, to give a final score used in the analysis between 0 and 100. The anxiety and depression scores collected from the HADS and used in analysis were the sum total across items (range 0 to 21).

Given the number of tests and testing sessions, missing data are inevitable. Table S1 therefore specifies the number of subjects per test and timepoint.

Total N (Tx_{90} nC/Tx_{90} C/Tx_{180} nC/Tx_{180} C)	BL-Pre	Pre-Post	Post-Fup6	Fup6-Fup12
FAT	57 (16/17/12/12)	65 (19/18/14/14)	31 (9/10/6/6)	19 (5/6/4/4)
WMFT	57 (16/17/12/12)	65 (19/18/14/14)	31 (9/10/6/6)	20 (5/7/4/4)
MAL	58 (16/17/13/12)	65 (19/18/14/14)	34 (10/10/8/8)	23 (6/7/6/4)
NHPT	55 (15/17/12/11)	63 (18/18/14/13)	30 (9/10/6/5)	19 (5/7/4/3)
SIS	55 (15/16/12/12)	64 (18/18/14/14)	33 (9/10/8/6)	23 (7/7/5/4)
SF36	52 (15/14/12/11)	61 (18/17/13/13)	32 (9/10/8/5)	22 (7/7/5/3)
HADS	53 (15/15/12/11)	60 (18/17/12/13)	31 (9/10/7/5)	21 (6/7/5/3)
VAMS	53 (15/15/12/11)	60 (18/16/13/13)	32 (9/10/8/5)	22 (7/7/5/3)

Table S1. Numbers of patients completing each test for each time point. Grey box represents participants described in Table 1. Total N: total participants; (Tx_{90} nC/Tx_{90} C/Tx_{180} nC/Tx_{180} C): number of participants in each treatment group. FAT: Frenchay Arm Test; WMFT: Wolf Motor Function Test; MAL: Motor Activity Log; NHPT: Nine Hole Peg Test; SIS: Stroke Impact Score; SF36: Short Form 36; HADS: Hospital Anxiety and Depression Scale; VAMS: Visual Analogue Mood Score.

Reliable change index methodology

The RCI method is an analytical tool, which can be used to determine the statistical meaningfulness of test score differences in clinical samples. This method incorporates an adjustment for the test-retest reliability of the assessment measure (e.g. questionnaire) being employed. For each measure, participants' test score differences between assessment intervals are divided by the standard error of difference. This normalises these difference scores and transforms them into standardised values such that they can be subsequently interpreted through comparison to the z-score distribution. Resultant z-scores greater than ± 1.96 therefore indicate whether observed changes in participants' scores are significantly greater than what would have been expected by chance alone ($p < .05$). This technique is

sample-size independent and has the advantage over more traditional analytic techniques of being particularly adept at determining clinical deterioration as well as improvement. Moreover, as this method accounts for temporal instability in the assessment measure, it enables adjustment to be made for baseline instability in participants' test scores. The standard error of difference is usually calculated using previously established normative statistics for the assessment tool in question (e.g.[34]). However, we applied the RCI method in a slightly more novel but equally appropriate way by using scores from the baseline to pre-therapy assessment interval to determine the standard error of difference. Preliminary power analyses confirmed the sufficiency of the sample size for this task. As a consequence, the RCI methodology employed in this study represented a transformation of the observed data into standardised difference scores (between pre, post and follow up) with adjustment for the changes seen from baseline to the start of therapy. This ensured that any changes identified during and after the intervention were more directly attributable to the treatment itself. Raw outcome scores can be seen in Table S2 for reference.

	Baseline	Pre-CIMT	Post-CIMT	6 Mth	12 Mth
FAT	1.39±0.22	1.48±0.21	2.03±0.21	1.87±0.33	2.65±0.38
WMFT TT	40.19±5.61	37.31±5.19	30.50±4.93	29.13±7.16	21.11±6.42
WMFT FAS	3.66±0.18	3.69±0.16	4.18±0.17	4.11±0.22	4.67±0.26
MAL QoM	0.75±0.10	0.79±0.10	1.62±0.13	1.45±0.19	1.71±0.21
MAL AoU	0.70±0.11	0.78±0.10	1.65±0.15	1.47±0.21	1.69±0.22
NHPT Small	4.62±1.15	4.84±1.13	6.83±1.25	6.97±1.86	9.92±2.27
NHPT Large	6.25±1.09	7.92±1.24	8.50±1.24	8.47±1.92	12.04±2.11
SIS Total	61.03±1.45	61.48±1.50	68.39±1.48	68.35±1.70	67.02±2.33
SIS Physical	50.47±1.65	49.79±1.76	58.05±1.72	58.59±2.11	57.75±2.71
SF-36 Physical	56.14±2.09	57.41±1.92	59.07±1.88	61.28±2.32	60.13±2.61
SF-36 Mental	66.77±2.21	68.01±1.97	69.38±2.00	71.85±2.48	71.76±2.83

HADS Anxiety	5.58±0.41	5.53±0.41	4.70±0.40	4.48±0.45	5.34±0.70
HADS Depression	5.57±0.41	5.39±0.43	5.16±0.48	4.45±0.47	4.22±0.64
VAMS Positive	42.12±1.54	43.83±1.25	42.45±1.43	43.27±1.64	43.73±1.87
VAMS Negative	49.73±0.99	49.97±0.98	50.45±1.21	48.27±1.61	50.19±1.43

Table S2. Raw outcome measure scores across all participants for each time point (Mean ±SEM). FAT: Frenchay Arm Test; WMFT: Wolf Motor Function Test; TT: Time Taken; FAS: Functional Ability Score; MAL: Motor Activity Log; QoM: Quality of Movement; AoU: Amount of Use; NHPT: Nine Hole Peg Test; SIS: Stroke Impact Score; SF36: Short Form 36; HADS: Hospital Anxiety and Depression Scale; VAMS: Visual Analogue Mood Score.

Analysis and statistics

To account for dropouts in the follow-up period, factorial ANOVAs with CONSTRAINT condition (C/nC) and TRAINING INTENSITY (T_{180}/T_{90}) were calculated for all patients completing the pre and post measures, as well as the subgroup of patients who completed the 6 month and 12 month follow-up measures respectively.

2. **Results:**

Comparison of the effect of training intensity and constraint on outcome

Follow-up:

Of the scales that demonstrated change over the follow-up period, only WMFT FA differed between the groups, with patients who had not used a CONSTRAINT improving more than those who had used a CONSTRAINT between Fup6 and Fup12 ($F(1,19) = 4.6$, $p = .047$, $\eta_p^2 = .22$). This is more pertinent when it seems from the figure (Figure 3) that the difference may be caused by the lack of improvement seen in the Tx_{90} C group, and when split into the four treatment groups, there are only four to seven participants in each group, making it difficult to be sure of the findings.

Differences were further observed for WMFT-TT, NHPT_L, SIS Physical and SF36 Physical at follow-up despite these scales showing no difference across all participants (Figures 1 and 2, and Table 2). From post-treatment to Fup6 there was a greater improvement in WMFT TT in those who had been in the Tx_{90} group compared to those in the Tx_{180} group ($F(1,30) = 4.2$, $p = .049$, $\eta_p^2 = .14$), and an interaction between CONSTRAINT and TRAINING INTENSITY for the SIS Physical scale ($F(1,32) = 6.1$, p = ..20, $\eta_p^2 = .17$). This interaction was caused by with greater improvement in the Tx_{90} nC and Tx_{180} C groups compared to the other groups. From Fup6 to Fup12 there was a greater improvement in those who did not use a CONSTRAINT for NHPT_L (nC>C; $F(1,18) = 7.7$, $p = .014$, $\eta_p^2 = .22$), and an effect of TRAINING INTENSITY (Tx_{180} improving more compared to Tx_{90}) on the SF36 Physical scale ($F(1,21) = 5.8$, $p = .026$, $\eta_p^2 =.22$). However, it should be borne in mind that the majority of the differences between groups in follow up are slight, in small sample sizes, and in scales that showed no overall change across groups.
